# Alleviation of Irritable Bowel Syndrome-Like Symptoms and Control of Gut and Brain Responses with Oral Administration of *Dolichos lablab* L. in a Mouse Model

**DOI:** 10.3390/nu10101475

**Published:** 2018-10-10

**Authors:** Eunho Chun, Soojung Yoon, Amna Parveen, Mirim Jin

**Affiliations:** 1 College of Medicine, Gachon University, Incheon 21999, Korea; iknow1004@hanmail.net (E.C.); sjyoon0725@gmail.com (S.Y.); 2 College of Pharmacy, Gachon Institute of Pharmaceutical Science, Gachon University, Incheon 21936, Korea; amnaparvin@gmail.com; 3 Department of Pharmacognosy, College of Pharmacy, Government College University Faisalabad, Faisalabad 38000, Pakistan; 4 Department of Health Science and Technology, GAIHST, Gachon University, Incheon 21936, Korea

**Keywords:** irritable bowel syndrome, *Dolichos lablab* L., zymosan-induced IBS, mouse model, gut–brain axis, colonic inflammation, pain, anxiety

## Abstract

Irritable bowel syndrome (IBS) is a common gastrointestinal disorder manifesting as unexplained abdominal pain and bowel habit changes. The pathogenesis of post-infectious IBS is associated with gut–brain axis dysfunction, including low-grade colonic inflammation and anxiety-related long-term brain changes. This study analyzed the efficacy of a standardized extract of *Dolichos lablab* L. extract (DL), a bean species, in an IBS mouse model resembling post-infectious, diarrhea-dominant IBS. Using a zymosan-induced animal IBS model, we found that oral administration of DL significantly attenuated zymosan-induced increases in colonic macroscopic scores and minimized weight loss without affecting food intake. In the DL-treated mice, the mast cell count and tumor necrosis factor-α level in the colon markedly decreased, similar to results in sulfasalazine-treated mice and in mice with lipopolysaccharide-stimulated bone marrow-derived mast cells. The number of visceral pain-related behaviors was much lower in the DL-treated mice. Anxiety-like behaviors significantly improved, comparable to that after treatment with amitriptyline. The c-Fos expression level in the prefrontal cortex was significantly reduced. Our data suggest that DL could be beneficial for treating IBS by acting on the gut and brain.

## 1. Introduction

Irritable bowel syndrome (IBS) is an extremely common bowel disorder that manifests as unexplained abdominal pain or discomfort and bowel habit changes of diarrhea, constipation, or alternating patterns of the two [1]. Current therapy for this illness focuses on relieving symptoms. The development of adequate medications for IBS has been hampered by a poor understanding of the complex pathophysiology involved in the multiple causative pathways [2]; however, gut–brain axis dysfunction is considered to be one of the most logical pathologic mechanism [3]. Compared to healthy subjects, patients with IBS generally have visceral hypersensitivity, which is defined by an overly sensitive recognition of local stimuli in the intestinal walls. This hypersensitivity contributes to uncomfortable sensations and peripheral pain [4], and it might be caused by dysregulated bi-directional communication between the enteric nervous system (ENS) and central nervous system (CNS), the so-called “gut–brain axis”. Stresses in the gut affect the function of the ENS and immune system, which affects CNS function. Conversely, gastrointestinal (GI) activity can be altered in response to physiological stress [5]. This is because signaling along the gut–brain axis is regulated by a reflex network of afferent intestinal fibers traveling to the CNS structures, as well as efferent projections from the CNS to the smooth muscle in the intestinal walls [3]. In the intestine of patients with post-infectious IBS, which generally develops after acute and severe gastroenteritis [6], low-grade inflammation induced by mast cells is thought to be responsible for the dysregulation [7]. These patients have a considerable increase in the number of mast cells in the colonic mucosa compared to healthy subjects [8,9]. Mast cell mediators activate the ENS, which leads to increases in the severity and frequency of abdominal pain [10,11]. The continuous triggers from primary sensory afferent neurons entering the CNS further induce long-term changes in the brain areas that are responsible for pain processing, and discomfort and emotional responses [12]. It has been suggested that the brain of patients with IBS may respond significantly to external or internal stressors [13], and functional brain imaging studies have indicated that colorectal stimulation may possibly activate the prefrontal cortex, anterior cingulate cortex, and other limbic brain regions [14]. Therefore, patients with IBS frequently exhibit depression and anxiety [15], which seems to negatively affect GI function.

The seed of *Lablab purpureus*, commonly known as Semen *Dolichos lablab* L., belongs to the family *Fabaceae* [16,17,18]. *D. lablab* L. has traditionally been used for treating GI disorders in China [19] and Korea [20], and the cooked pods of *D. lablab* L. are consumed for treatment of diarrhea, nausea, vomiting, and poor appetite in India [21]. However, no studies have investigated its use for treating IBS.

In this study, we analyzed the efficacy of a standardized *D. lablab* L. extract (DL) in an IBS mouse model resembling post-infectious and diarrhea-dominant IBS. The injection of zymosan into the colon once daily for 3 days results in the occurrence of IBS-like symptoms; initially mild to moderate diarrhea occurs, and pain and anxiety are observed thereafter [22,23,24,25]. Here, we aimed to determine whether DL has the potential to be used as a beneficial agent for treating IBS.

## 2. Materials and Methods

### 2.1. Preparation of D. lablab L.

The seeds of *D. lablab* L. were obtained from UniGEN Co., Ltd. (Cheonan-si, Korea; batch number 1701) and identified in Gachon University by one of the authors (A.P.). To prepare the DL, the seeds were washed and dried at room temperature. One kilogram of dried seeds was ground, and the extraction was performed using 10 L of 50% ethanol at 80 °C. The extracts were passed through filter paper (Whatman, Florham Park, NJ, USA) and concentrated in an R-220SE rotary evaporator (BUCHI, Flawil, Switzerland) at 55–65 °C under reduced pressure, and then dried in a SC-VAC400 vacuum dryer (Sungchan, Pocheon-si, Gyeonggi-do, Korea).

### 2.2. Standardization of DL

For standardization of DL, we set trigonelline, aspartic acid, and arginine as standard components. For trigonelline analysis, high-performance liquid chromatography (HPLC) was performed with a Waters system (Waters Corp., Milford, MA, USA), which was connected to a separation module (e2695), and a photodiode detector was used. For quantification, we used the analytical column Xbridge HILIC (4.6 × 150 mm, 5 µm; Waters Corp.) with the detection wavelength set at 264 nm. The mobile phase comprised two solutions: solvent A, acetonitrile, and solvent B, 0.1% phosphoric acid. The gradient elution used was as follows: 0 min → 10 min, 50% → 60% A and 10 min → 20 min, 60% → 70% A. The column was cleaned for 10 min with 90% A. The re-equilibrium time was 5 min. The flow rate was set at 0.8 mL/min, column temperature was kept at 30 °C, and injected volume was 10 µL. The extract powder was resuspended in ethanol (1 mg/mL) and filtered using a 0.4-µm membrane. Trigonelline (Pubchem CID: 5570) was used as a standard compound at a concentration of 1 mg/mL.

To analyze the amino acids, the extracted powder was resuspended in methanol, prepared at a final concentration of 1 mg/mL, and filtered through a 0.4-µm membrane filter before HPLC analysis. Aspartic acid and arginine were used as standard biomarkers at a concentration of 20 µg/mL. Before standardization, derivatization of all samples was performed by adding 10 µL of a derivatization solution to each sample. The derivatization solution consisted of a borate buffer (0.4 M, pH 9.5, 900 µL), o-phthalaldehyde (100 UL, 10 mg/100 µL MeOH), and β-mercaptoethanol (25 µL). The Waters system was used for HPLC, and it was connected with a fluorescence detector (2475), which was used for amino acid analysis. Amino acid estimation was done as prescribed by using a C18 column (250 mm × 4.6 mm, 5 µm). The mobile phase comprised acetonitrile containing 2% tetrahydrofuran (THF) and a sodium phosphate buffer (0.04 M, pH 7.0) containing 2% THF. The mobile phase was run in a gradient as follows: 0 min → 30 min and 15% → 25% A. The column was cleaned for 5 min with 85% A and returned to its initial condition.

### 2.3. Animal Experiments

Seven-week-old male C57BL/6 mice were purchased from Daehan Biolink, Inc. (Chungbuk, Korea). All animal experiments were performed in accordance with the guidelines of the Gachon University Animal Care and Use Committee (study approval number: LCDI-2017-0053). The mice were acclimated for 1 week. All mice were randomly divided into seven groups (*n* = 6/group) as follows: naïve group, control group (zymosan injection), DL group (100 mg/kg, 200 mg/kg, or 400 mg/kg), amitriptyline group (AMT; 30 mg/kg, Sigma-Aldrich, St. Louis, MO, USA), and sulfasalazine group (SSZ; 30 mg/kg, Sigma-Aldrich). To induce IBS, a 0.1-mL suspension of zymosan (30 mg/mL in phosphate-buffered saline (PBS); Sigma-Aldrich) was administered into the colons of all groups of mice except the naïve group for 3 consecutive days [25]. The naïve mice had PBS injected into their colons. Three hours after the zymosan injection, DL (100 mg/kg, 200 mg/kg, or 400 mg/kg), AMT (30 mg/kg), and SSZ (30 mg/kg) were orally administered with PBS as the vehicle using a 22-gauge feeding needle. On day 4 or 15, mice were sacrificed by cardiac puncture under isoflurane anesthesia.

### 2.4. Macroscopic Scoring of Zymosan-Induced Colon Changes

The colon weights of mice were measured after removing the fecal contents, and colon lengths were determined from the aboral end of the cecum to the anus. Stool conditions were scored by three researchers in a blind manner. The individual scores of colon weight (increase vs. naïve), colon length (decrease vs. naïve) (score 0, <5%; 1, 5–14%; 2, 15–24%; 3, 25–35%; and 4, >35%), and stool score (0: normal; 1: loose/moist; 2: amorphous/sticky; and 3: diarrhea) were graded. The total macroscopic score index of the severity of colonic changes was defined as the sum of the individual macroscopic score indices for each colon, with 0 representing normal and 11 being maximally affected, as described by Kimball et al. [26].

### 2.5. Body Weight Changes and Food Intake

Body weights were measured on days 1, 4, 7, 10, and 14. Food intake was estimated to be the difference between the amounts of food remaining in the feeder on day 14 and the amount given on day 1 [26].

### 2.6. Histological Examination

Tissues were obtained from the large intestine of the mice in each group (*n* = 6) after sacrifice. The sections were fixed and embedded in paraffin, cut to a thickness of 4 µm, and stained with hematoxylin and eosin (H&E) or toluidine blue to detect infiltrated inflammatory cells or mast cells, respectively. The cells were observed under a visible-light microscope (Nikon, Tokyo, Japan) at a magnification of 200×. The mucosa thickness was measured from the submucosa to the mucosa epithelium. Infiltrated inflammatory cells and mast cells in the mucosa were counted with ImageJ software (National Institutes of Health, Rockville, MD, USA).

### 2.7. Polymerase Chain Reaction (PCR)

Total RNA was isolated by using the Trizol reagent (Invitrogen, Carlsbad, CA, USA), and the RNA sample was used for cDNA synthesis using a PrimeScript RT reagent kit (TaKaRa, Shiga, Japan). The mRNA of tumor necrosis factor (TNF)-α and glyceraldehyde-3-phosphate dehydrogenase (GAPDH) were quantified using a SimpliAmp Thermal Cycler (Applied Biosystems, Foster City, CA, USA) with Accupower PCR PreMix (Bioneer, San Francisco, CA, USA). The following primers were used: mouse TNF-α forward; 5′-CTC CCA GGT TCT CTT CAA GG-3′, reverse; 5′-TGG AAG ACT CCT CCC AGG TA-3′, mouse GAPDH forward; 5′-GCT TGT CAT CAA CGG GAA G-3′, reverse; and 5′-TTG TCA TAT TTC TCG TGG TTC A-3′. The polymerase chain reaction was performed at 94 °C for 5 min, at 95 °C for 20 s, at 57 °C for 20 s, and at 72 °C for 45 s for 32 cycles.

### 2.8. Murine Bone Marrow-Derived Mast Cells Culture

Bone marrow-derived mast cells (BMMCs) were derived from femoral bone marrow cells of 6-week-old C57BL/6 mice and cultured with interleukin (IL)-3 (10 ng/mL) for 3 weeks and then cultured in roswell park memorial institute (RPMI) 1640 medium (Lonza, Allendale, NJ, USA) with IL-3 plus a stem cell factor (10 ng/mL) for 3 weeks. Complete differentiation takes 4–6 weeks. The cell consisted of >92% mast cells determined by fluorescence-activated cell sorting (FACS) analysis (LSR II, BD Biosciences, San Jose, CA, USA) for c-Kit expression (Invitrogen).

### 2.9. Enzyme-Linked Immunosorbent Assay (ELISA)

BMMCs (1 × 10^6^ cells/mL) were treated with DL with various concentrations followed by stimulation with lipopolysaccharide (LPS) (5 µg/mL) for 6 h. The levels of TNF-α were determined using a commercially available ELISA kit (R&D, Minneapolis, MN, USA).

### 2.10. Pain-Related Behavior Test

Visceral pain-related behaviors were counted, and the total number of visceral pain-related behaviors was recorded over 10 min, as previously described [23,27].

### 2.11. Anxiety-Related Behavior Test

The elevated plus maze (EPM) test was performed as previously described [23]. The recordings were analyzed using a video tracking software (SMART 3.0; Panlab S.I., Barcelona, Spain). For the open field test (OFT), the open field arena (30 × 30 cm) was constructed from acrylic sheets, and each mouse was placed in the center of the field. The mice were individually transferred to the test field, and their behaviors were recorded for 30 min. The recordings were analyzed using the same software as in the EPM, as described by Zhang et al. [25].

### 2.12. Immunofluorescence Analysis (IF)

Animals were respiratory anesthetized continuously with isoflurane and transcardially perfused with 4% paraformaldehyde (PFA) in PBS. Mice brains were pre-fixed with 4% PFA overnight and kept overnight in 20% sucrose in PBS. The brains were frozen and sectioned into 20-µm thick serial coronal sections using an optimal cutting temperature compound. The frozen section was fixed with 4% paraformaldehyde in PBS for 20 min at room temperature, permeabilized with 0.1% triton X-100 in PBS, and blocked with 2% bovine serum albumin for 1 h at room temperature. Sections were incubated overnight with the c-Fos antibody (1:50, Santa Cruz Biotechnology, Santa Cruz, CA, USA) at 4 °C, washed with PBS for 10 min each, and incubated with fluorescein isothiocyanate-conjugated AffiniPure goat anti-mouse immunoglobulin G (IgG) (H + L) (1:200, Jackson ImmunoResearch, Inc., West Grove, PA, USA) for 1 h at room temperature. C-Fos expression was determined by confocal microscopy (LSM T-PMT; Zeiss, Oberkochen, Germany), and ZEN software (Zeiss). ImageJ software (National Institutes of Health, Rockville, MD, USA) was used to quantify the fluorescence intensity of c-Fos expression. The counts of c-Fos expression in the brain regions were represented as the average number of positively stained cells in a minimum of six sections per brain region.

### 2.13. Western Blotting

Brain samples were homogenized; equal amounts (40 µg) of proteins were separated by sodium dodecyl sulfate–polyacrylamide gel electrophoresis and transferred to nitrocellulose membranes (Amersham Biosciences, Piscataway, NJ, USA). The membranes were probed overnight with antibodies specific to c-Fos (Santa Cruz Biotechnology, Inc.), as previously described [23]. The band density was compared using β-actin and measured using ImageJ software.

### 2.14. Statistical Analysis

All data are expressed as a mean ± standard deviation (SD). One-way analysis of variance (ANOVA) was performed using SPSS software (IBM-SPSS Inc., Chicago, IL, USA) to analyze the differences between the groups. Multiple group comparisons were performed using one-way ANOVA, followed by post hoc Tukey tests. The differences that had *p* < 0.05 were considered statistically significant.

## 3. Results

### 3.1. Standardization of DL

**Figure 1:**
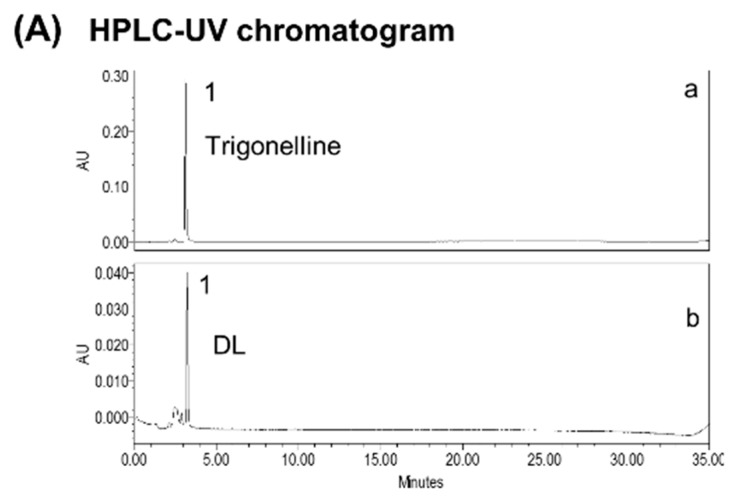

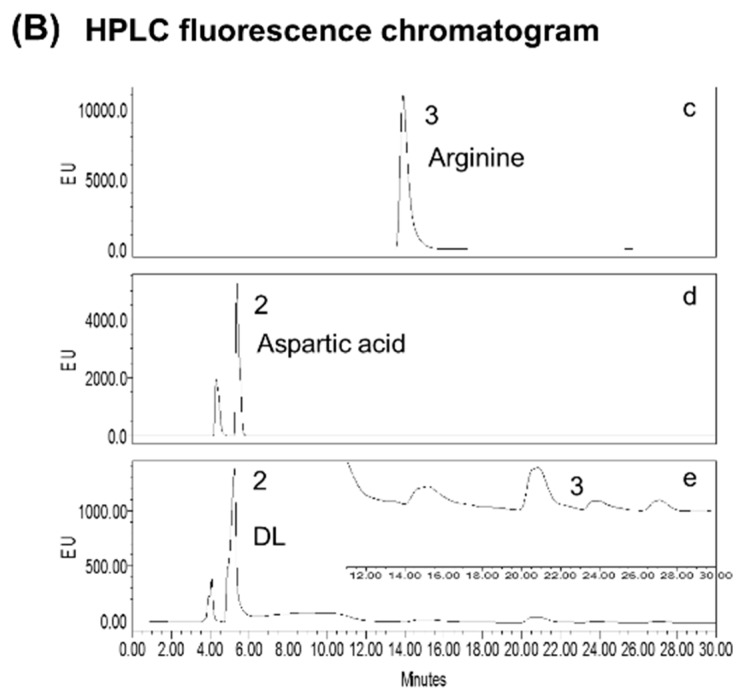
HPLC chromatogram for determining DL. (**A**) HPLC-UV chromatogram of trigonelline (1, a) and DL (b) at 264 nm. (**B**) HPLC fluorescence chromatogram of arginine (3, c) and aspartic acid (2, d) as standard components, and DL (e). HPLC, high-performance liquid chromatography; DL, *D. lablab* L. extracts.

For standardization of the DL, we set trigonelline, arginine, and aspartic acid as marker contents using HPLC. Figure 1 shows a chromatogram of DL and the standard content. The linearity of each compound was calculated at three concentrations. The contents of trigonelline, arginine, and aspartic acid were 1.20 ± 0.068, 9.4 ± 0.05, and 0.009 ± 0.00 µg/100 µg of DL.

### 3.2. Effects of DL on the Macroscopic Score of Zymosan-Induced Colon Changes

**Figure 2:**
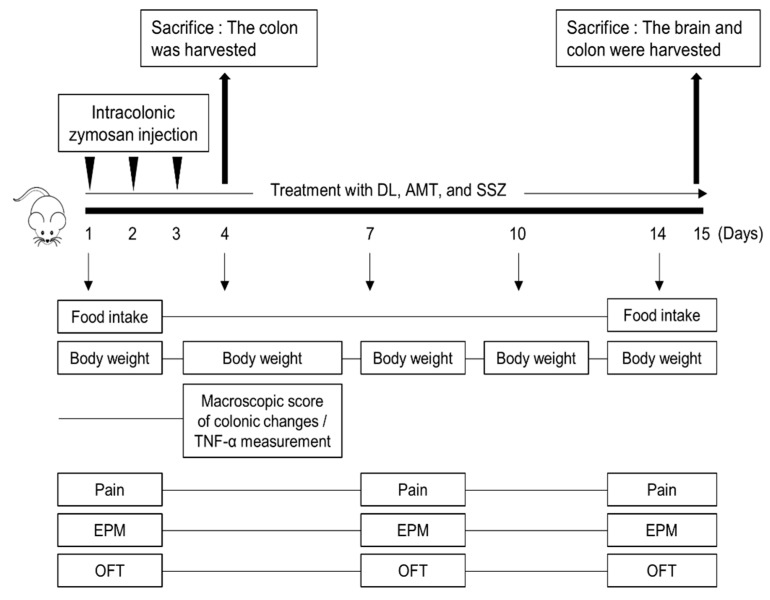
Animal experimental scheme. DL, *Dolichos lablab* L. extract; AMT, amitriptyline; SSZ, sulfasalazine; EPM, elevated plus maze; OFT, open field test.

First, we examined the effects of DL on the macroscopic changes in the zymosan-injected mice colons. The experimental scheme in Figure 2 shows DL (100 mg/kg, 200 mg/kg, and 400 mg/kg) and the two drugs defined as positive controls, including AMT (30 mg/kg), an antidepressant [28], and SSZ (30 mg/kg), an anti-inflammatory medication [29], both of which are currently used to treat patients with IBS. The colon length, weight, and stool condition of control mice were measured and compared to those of naïve mice. The zymosan injections induced distal colon shortening in the control mice compared to the naïve mice, which indicated colitis. However, DL administration increased colon lengths in the DL group compared to the control (DL100 [100 mg/kg of DL]: 8.9 ± 0.25 cm, *p* < 0.01; DL200 [200 mg/kg of DL]: 8.98 ± 0.37 cm, and DL400 [400 mg/kg of DL]: 8.96 ± 0.37 cm, *p* < 0.001). Furthermore, colon weights were significantly increased in the control mice compared to the naïve mice; however, DL significantly decreased the colon weights (DL100: 0.64 ± 0.07 g, DL200: 0.62 ± 0.04 g, and DL400: 0.62 ± 0.10 g, all *p* < 0.05) such that the weights of the DL mice approached those of the naïve mice. SSZ significantly suppressed the zymosan-induced increase in the macroscopic score, and AMT reduced colitis and diarrhea, although to a lesser extent than SSZ (Table 1). The naïve mice defecated dark brown rigid feces, whereas the control mice had watery, sticky masses of bright brown or yellow-colored feces, indicating moderate diarrhea. The stool score increased in the control mice compared to the naïve mice, but DL administration significantly reduced the stool score of the DL mice compared to the control mice (DL100: 1.30 ± 0.69; DL200: 1.00 ± 0.50, and DL400: 1.25 ± 0.73; all *p* < 0.01; Table 1). The macroscopic score (sum of the colon weight, length, and stool score) was significantly increased by injections of zymosan and indicated low-to-moderate colitis and diarrhea (Table 1). The oral administration of DL decreased the macroscopic score of DL mice compared to the control mice, suggesting that DL might suppress zymosan-induced mild colitis and diarrhea.

### 3.3. Effects of DL on Body Weight Changes and Food Intake

**Figure 3:**
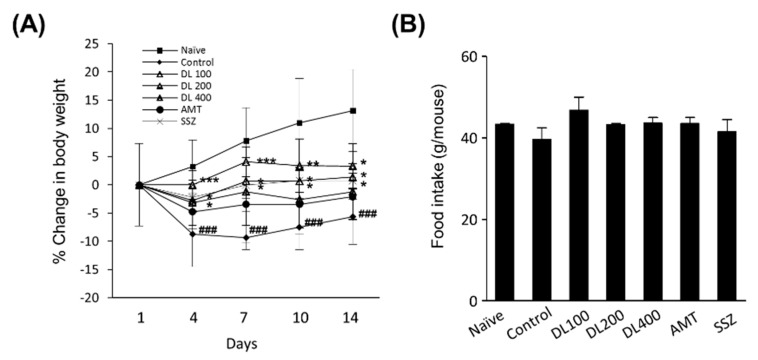
Effects of DL on the body weight and food intake of zymosan-induced irritable bowel syndrome (IBS) mice. (**A**) Body weight and (**B**) food intake were measured on the indicated days. Data are presented as a mean ± standard deviation (*n* = 6, one-way ANOVA; ### *p* < 0.001 vs. naïve; * *p* < 0.05, ** *p* < 0.01, *** *p* < 0.001 vs. control). DL, *D. lablab* L. extracts; AMT, amitriptyline; SSZ, sulfasalazine.

Next, we examined the effects of DL on zymosan-induced decreases in body weight (Figure 3A). Mean body weights were initially comparable across the seven groups, but injections of zymosan were associated with significantly decreased body weights on days 4, 7, 10, and 14 in the control mice compared to the naïve mice. However, oral DL administration suppressed the loss of body weight changes on day 4 (Figure 3A). SSZ administration significantly improved the body weights on days 4 (SSZ: −2.20 ± 2.4%, *p* < 0.05) and 7 (SSZ: 0.00 ± 4.8%, *p* < 0.05), but AMT did not attenuate the weight loss (Figure 3A). No difference was observed in food intake among the groups (Figure 3B). These results suggest that DL attenuated the decreases in body weight without affecting food intake in mice with zymosan-induced IBS.

### 3.4. Effects of DL on Zymosan-Induced Colonic Inflammation

**Figure 4:**
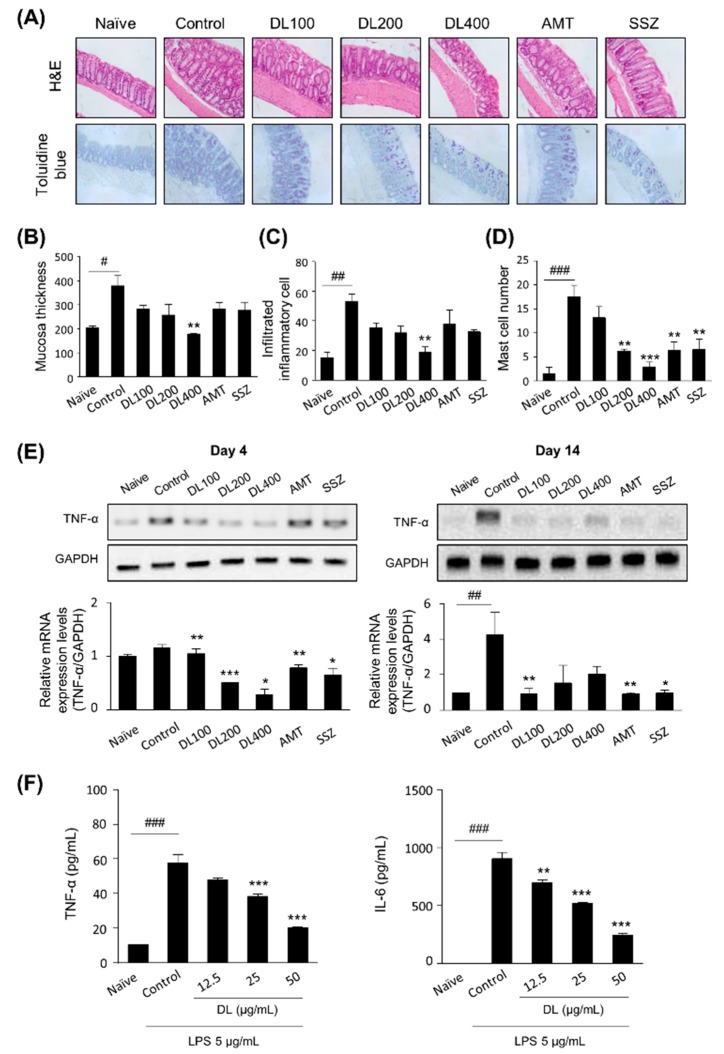
Effects of DL on colonic inflammation. Formalin-fixed colons were stained with (**A**) H&E and toluidine blue. Cells in the colon were visualized under a visible-light microscope at a magnification of 200×. (**B**) The mucosa thickness were measured and (**C**) infiltrated inflammatory cells and (**D**) mast cells were counted by ImageJ software. (**E**) TNF-α expression levels and colon histology in zymosan-induced IBS mice. The level of TNF-α mRNA in the colon on days 4 and 14 were determined by reverse transcription polymerase chain reaction (RT-PCR). (**F**) Bone marrow-derived mast cells (BMMCs) were treated with various concentrations of DL for 1 h followed by stimulation with LPS for 6 h. Levels of TNF-α were determined by enzyme-linked immunosorbent assay (ELISA). The images represent one of three or six independent experiments. Data are presented as a mean ± SD (*n* = 6, one-way ANOVA; ## *p* < 0.01, ### *p* < 0.001 vs. naïve; * *p* < 0.05, ** *p* < 0.01, *** *p* < 0.001 vs. control). H&E, hematoxylin and eosin; DL, *D. lablab* L. extracts; AMT, amitriptyline; SSZ, sulfasalazine; TNF-α, tumor necrosis factor-α; LPS, lipopolysaccharides; GAPDH, glyceraldehyde-3-phosphate dehydrogenase.

We examined the effect of DL on colonic inflammation, and histological examinations of zymosan-injected colons by H&E staining showed increased epithelial thicknesses [26] and submucosal inflammatory cell infiltrations, indicating low-grade to moderate-grade inflammation. However, in DL-treated mice, there were significant dose-dependent decreases in colon wall thicknesses and inflammatory cell infiltrations (Figure 4A–C). Toluidine blue staining revealed that the number of mast cells was highly increased and associated with inflammatory foci in the control group compared with the naïve group (Figure 4D). The number of infiltrating mast cells was markedly diminished in the DL-treated mice, similar to the SSZ-treated mice (Figure 4C). The effects of DL on colonic inflammation were further examined by measuring the mRNA expression of TNF-α, a representative pro-inflammatory cytokine. The significantly increased levels of TNF-α mRNA were observed in the colons of the control mice compared to those of the naïve mice; however, the expression levels decreased in the DL group compared to the control group on days 4 and 14 (Figure 4E). Data from BMMCs indicated that DL treatment significantly decreased LPS-induced TNF-α production in a dose-dependent manner (Figure 4F). These data suggest that DL might have inhibitory effects on GI inflammation in mice with zymosan-induced IBS.

### 3.5. Effects of DL on Visceral Pain-Related Behaviors

**Figure 5:**
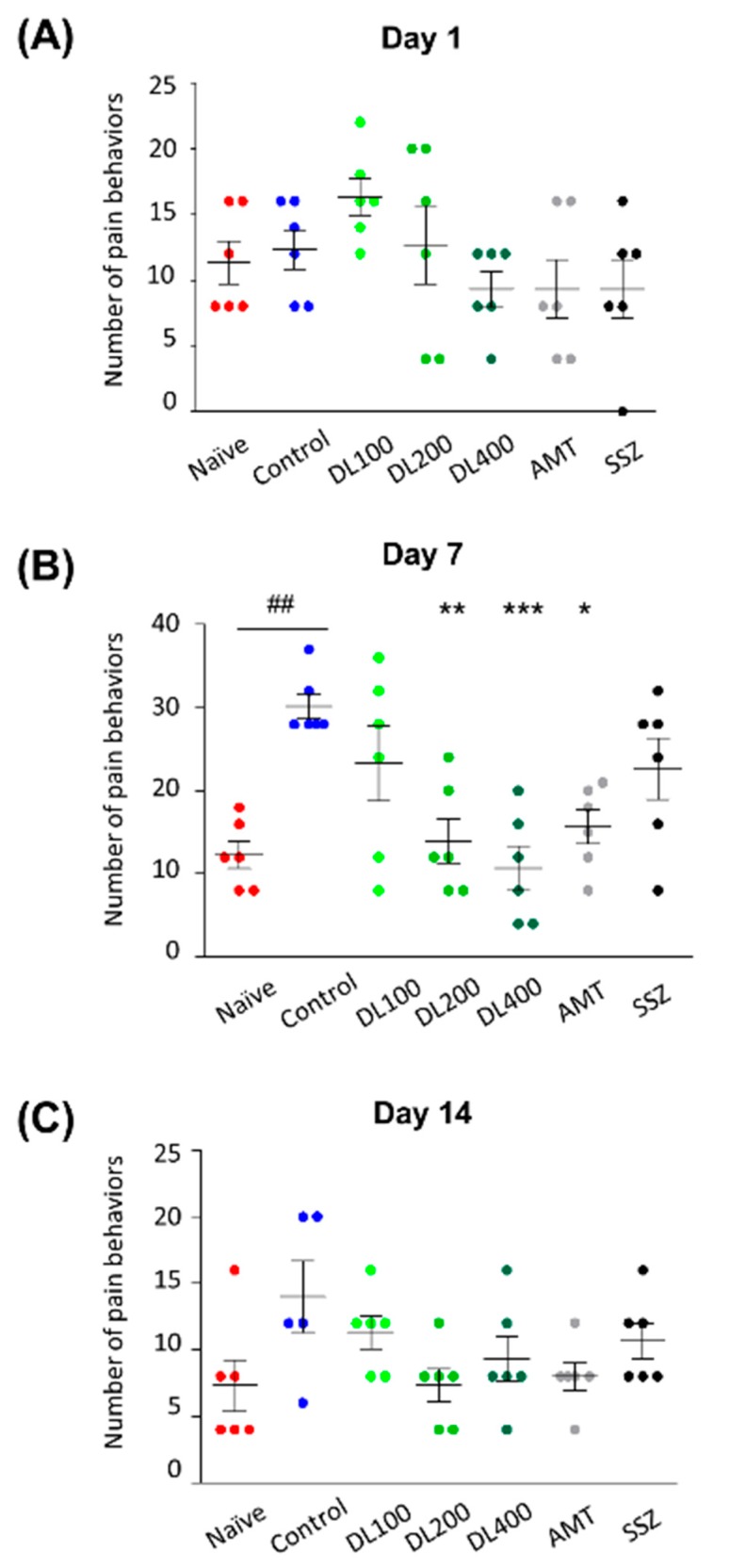
Effects of DL on visceral pain-related behaviors in zymosan-induced IBS mice. (**A**) Day 1, (**B**) day 7, and (**C**) day 14 according to the methods described in the Materials and Methods. Data are presented as a mean ± standard deviation (*n* = 6, one-way ANOVA; ## *p* < 0.01 vs. naïve; * *p* < 0.05, ** *p* < 0.01, *** *p* < 0.001 vs. control). DL, *D. lablab* L. extracts; AMT, amitriptyline; SSZ, sulfasalazine.

We investigated the effects of DL on visceral pain. We did not observe any significant pain-related behaviors in the control mice compared to the naïve mice on day 1 (Figure 5A). However, on day 7, the control mice frequently showed pain-related behaviors including flattening the abdomen against the floor, licking of the abdomen, abdominal retraction, and whole-body stretching. The administration of DL or AMT (DL200: 14.00 ± 6.57, *p* < 0.01; DL400: 10.67 ± 6.53, *p* < 0.001; AMT: 15.67 ± 5.01, *p* < 0.05) (Figure 5B), but not SSZ, decreased the frequency and intensity of pain-related behaviors on day 7. The behaviors did not persist through day 14 (Figure 5C). These results suggest that DL might suppress visceral pain in mice with zymosan-induced IBS.

### 3.6. Effects of DL on Anxiety-Like Behaviors

**Figure 6:**
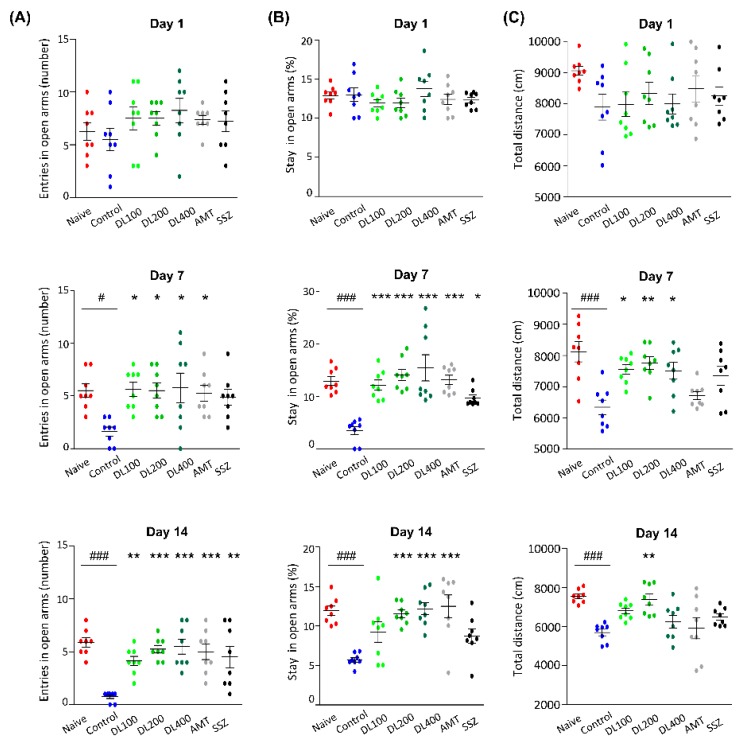
Effects of DL on anxiety-related behaviors in the EPM test and OFT. (**A**) The number of entries, (**B**) percentage of relative stay in open arms, and (**C**) travel distance in the OFT were measured on days 1, 7, and 14. Data are presented as a mean ± standard deviation (*n* = 8, one-way analysis of variance; # *p* < 0.05, ### *p* < 0.001 vs. naïve; * *p* < 0.05, ** *p* < 0.01, *** *p* < 0.001 vs. control). DL, *D. lablab* L. extracts; AMT, amitriptyline; SSZ, sulfasalazine; EPM, elevated plus maze test; OFT, open field test.

The number of entries and durations of stay in open arms were measured on days 1, 7, and 14 using the EPM test. We did not observe any significant decreases in locomotion on day 1, but there were dramatic reductions in the numbers of entry into open arms on days 7 and 14 (demonstrating low entry numbers, 1.63 ± 1.19 and 0.75 ± 0.46, respectively) in the control mice compared to the naïve mice. In the DL group, the number of entries into open arms markedly increased compared to the control group on day 14 (DL100: 4.13 ± 1.25, *p* < 0.01; DL200: 5.25 ± 1.04, *p* < 0.001; DL400: 5.50 ± 2.00, *p* < 0.001; Figure 6A). The durations of stay in open arms were significantly reduced in the control mice compared to the naive mice on days 7 (naïve: 12.92 ± 2.33% vs. control: 3.48 ± 2.22%, *p* < 0.001) and 14 (naïve: 11.93 ± 1.67% vs. control: 5.74 ± 0.82%, *p* < 0.001). The frequency of stay in open arms markedly increased in the DL group compared to the control on days 7 (DL100: 12.25 ± 2.72%, *p* < 0.001; DL200: 14.11 ± 3.00%, *p* < 0.001; DL400: 15.43 ± 7.08%, *p* < 0.001) and 14 (DL200: 11.56 ± 1.31%, *p* < 0.001; DL400: 12.20 ± 2.21%, *p* < 0.001) (Figure 6B). DL showed similar effects to AMT. In the OFT, the control mice showed a markedly decreased travel distance compared to naive mice; however, the travel distance of mice in the DL group increased on days 7 (DL100: 7550 ± 426.3 cm, *p* < 0.05; DL200: 7753 ± 577.5 cm, *p* < 0.01; DL400: 7505 ± 764.9 cm, *p* < 0.05) and 14 (DL200: 7399 ± 771.9%, *p* < 0.01), which was comparable to the finding in naive mice (Figure 6C). These data suggested that DL might control anxiety-like behaviors in mice with zymosan-induced IBS.

### 3.7. Effects of DL on c-Fos Protein Expression in the Brain

**Figure 7:**
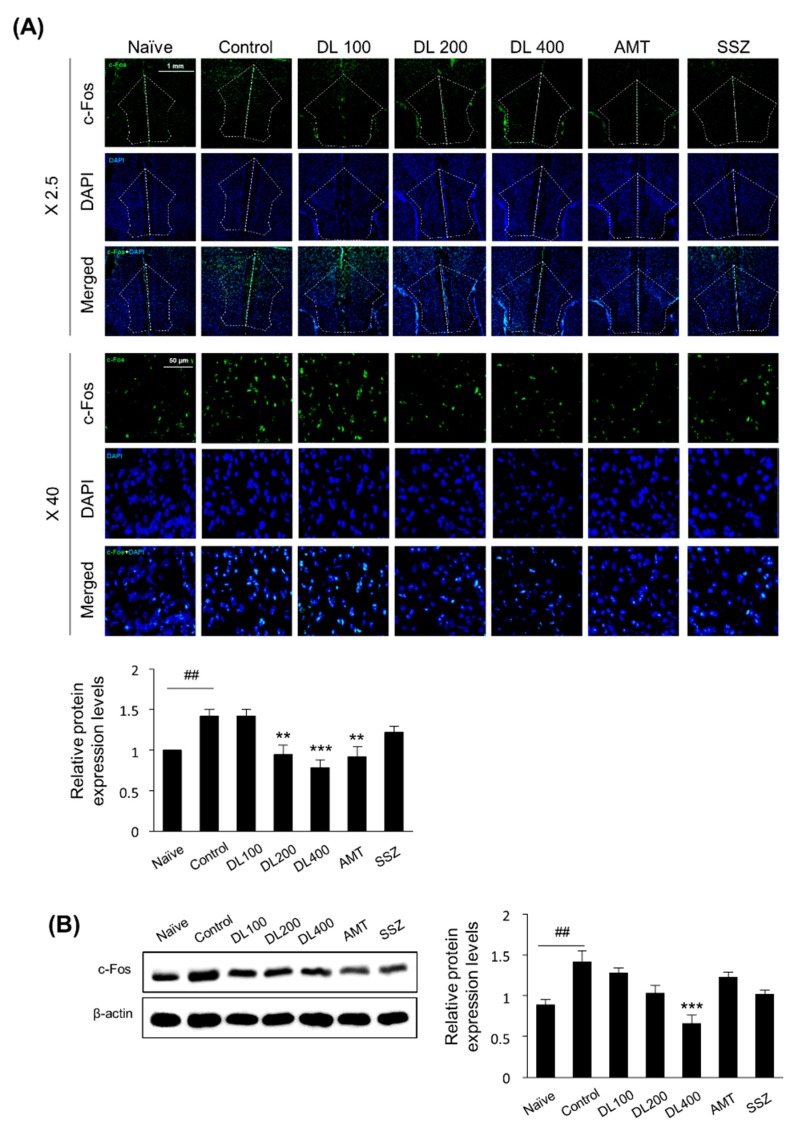
Effects of DL on c-Fos protein expression in the prefrontal cortex of zymosan-induced IBS mice. (**A**) IF staining was performed to determine c-Fos expression in the prefrontal cortex (1.78 mm before the bregma). The frozen sections were stained with c-Fos antibodies (green) and evaluated by confocal microscopy. The dashed line indicates the location of the prefrontal cortex. The protein level of c-Fos was quantified by ImageJ software. (**B**) Prefrontal cortex lysates were analyzed by Western blotting using c-Fos antibodies. Band density was analyzed by ImageJ software. β-actin was used as the loading control. Data are presented as a mean ± standard deviation (*n* = 4, one-way ANOVA; ## *p* < 0.01 vs. naïve; *** *p* < 0.001 vs. control). DL, *D. lablab* L. extracts; AMT, amitriptyline; SSZ, sulfasalazine; IF, immunofluorescence; DAPI, 4′,6-diamidino-2-phenylindole.

It has been reported that after intracolonic injections of zymosan, the c-Fos protein expression is highly increased in the brain areas responding to pain [25]. The c-Fos expressions in the prefrontal cortex determined by IF (Figure 7A) and Western blotting (Figure 7B) were significantly increased in the control mice compared to the naive mice. In the DL group there were dose-dependent reduced levels of c-Fos expressions (Figure 7A,B). These data suggested that DL might control neural responses in the brain against noxious stimuli from the gut.

## 4. Discussion

In this study, we assessed DL as a potential beneficial agent for IBS management using an animal model [25]. The zymosan injection induced colonic alterations such as diarrhea and low-to-moderate grade inflammation, which led to the attenuation of increased body weight. Zymosan also induced neural responses such as pain and anxiety-like behaviors, as previously reported [25], indicating the presence of gut–brain signaling.

First, we demonstrated that the oral administration of DL controlled colonic stress. Zymosan-induced colon length shortening and weight increases were significantly suppressed and diarrhea was inhibited. Further, the expression of the pro-inflammatory cytokine TNF-α was decreased in the colons of mice that received DL, indicating the anti-inflammatory properties of DL. The GI tract possesses an extensive immune system and a large neural network that facilitates communication between neurons and immune cells, including mast cells. The activation of mast cells located proximal to nerves has been attracting attention as a pathologic mechanism for the development of post-infectious IBS [30]. Newly synthesized cytokines, such as TNF-α and bioactive substances released from mast cells, are known to induce a series of neural effects, especially visceral pain. Conversely, nerves exposed to stress regulate mast cells by releasing various neuro-substances, which leads to barrier dysfunction and increased permeability [9]. It is noteworthy that there was a marked reduction in the number of mast cells and TNF-α expression in the DL-treated mice colons. In vitro assay using BMMCs further supported that DL has significant inhibitory effects on the pro-inflammatory cytokine expression in mast cells. Second, our data indicated that DL reduced the number of visceral pain-related behaviors. Third, the colonic injection of zymosan induced anxiety-like behaviors in mice resembling those observed in human IBS. Indeed, most patients with IBS show comorbid psychological symptoms, particularly anxiety and depression [31,32]. DL increased the number of entries and duration of stay in open arms using EPM and OFT, indicating anxiolytic effects. In an effort to provide molecular evidence, we analyzed c-Fos expression in the prefrontal cortex. C-Fos is a component of transcription factor AP-1 that regulates various genes that control synaptic responsiveness to a broad range of stimuli, including somatosensory stimulation, traumatic injury, seizures, and long-term potentiation [33]. It was reported that direct injections of zymosan into the colon as a noxious stimulus induced c-Fos expression in the forebrain, subcortex (including the amygdala), mid-brain, and brainstem, all of which are associated with pain and emotion [25,34]. Our data indicated that c-Fos expressions were significantly decreased in DL-treated mice, indicating that DL may suppress the input of harmful stimuli from the intestine to the brain or protect the brain from toxic challenges. Our study revealed that DL at a dose of 200 mg/kg was comparable to AMT at a dose of 30 mg/kg (Figure 5B and Figure 6B), a tricyclic antidepressant for managing pain and anxiety, and SSZ at a dose of 30 mg/kg (Table 1 and Figure 4A–D), an anti-inflammatory agent for colonic inflammation. Based on our data, it is plausible that the active component(s) of DL may directly enter the brain and protect the neural areas from noxious triggers produced by zymosan in the GI tract, resulting in the control of gut function. On the contrary, DL may directly suppress colonic inflammation and/or inhibit vagus nerve activation, which triggers neural effects on the GI system, resulting in the control of brain function. Simultaneous actions in the gut and brain cannot be excluded.

*D. lablab* L. is a rich source of secondary metabolites belonging to different classes of compounds including alkaloid (trigonelline), amino acids (aspartic acid and arginine), steroids (brassinolide, dolicholide, and dolichosterone), organic acids (palmitic acid and linoleic acid), and many other secondary metabolites [16,35,36]. The standardization of DL indicates that trigonelline, a member of the alkaloid group, has been found to be a main component according to HPLC-UV analysis. With an HPLC-florescence detector, aspartic acid has been found to be the main content along with arginine as a minor content. Trigonelline performs many biological activities that affect the brain, including but not limited to antinociception, the stimulation of dopamine release, and inhibition of the γ-aminobutyric acid (GABA) receptor response [37]. In addition, previous studies revealed that DL also contains amino acids such as arginine and aspartic acid [38]. Interestingly, it has been proven that dietary supplementation of amino acids such as aspartic acid and arginine facilitate mucosal healing and maintain normal intestinal physiology by reducing inflammation [39]. Further studies are warranted to identify active compound(s) and their mechanisms of action on the gut–brain axis at the molecular level.

Considering that the current treatment of IBS requires multi-drug therapy including antidiarrheal and anti-inflammatory agents, antidepressants, and analgesics, all of which should be simultaneously given to relieve the various symptoms of IBS, DL seems to be attractive and worthwhile as a potential agent for managing IBS.

## Figures and Tables

**Table 1:** Macroscopic score of zymosan-induced colon changes.

Group	Length Score	Weight Score	Stool Score	Macroscopic Score
**Naïve**	0.20 ± 0.40	0.00 ± 0.00	0.60 ± 0.58	0.80 ± 0.86
**Control**	1.40 ± 0.50 ###	3.25 ± 0.50 ###	2.95 ± 0.84 ###	6.95 ± 1.72 ###
**DL100**	0.00 ± 0.00 ***	1.25 ± 0.96	1.30 ± 0.69 **	2.30 ± 1.58 ***
**DL200**	0.00 ± 0.00 ***	1.00 ± 0.82 *	1.00 ± 0.50 **	1.80 ± 1.04 ***
**DL400**	0.00 ± 0.00 ***	1.25 ± 0.96	1.25 ± 0.73 **	2.25 ± 1.24 ***
**AMT**	0.00 ± 0.00 ***	2.25 ± 0.96	1.45 ± 0.82 *	3.25 ± 1.79 **
**SSZ**	0.40 ± 0.50 **	1.00 ± 0.82 *	1.45 ± 0.41 *	2.65 ± 1.42 ***

Colonic changes including the measured colon length, colon weight, stool score, and macroscopic score indices were analyzed as described in the Materials and Methods. Data are presented as a mean ± standard deviation (*n* = 6, one-way ANOVA; ### *p* < 0.001 vs. naïve group; * *p* < 0.05, ** *p* < 0.01, *** *p* < 0.001 vs. control). DL, *D. lablab* L. extracts; AMT, amitriptyline; SSZ, sulfasalazine.
